# SAAFEC: Predicting the Effect of Single Point Mutations on Protein Folding Free Energy Using a Knowledge-Modified MM/PBSA Approach

**DOI:** 10.3390/ijms17040512

**Published:** 2016-04-07

**Authors:** Ivan Getov, Marharyta Petukh, Emil Alexov

**Affiliations:** Computational Biophysics and Bioinformatics, Physics Department, Clemson University, Clemson, SC 29634, USA; igetov@clemson.edu (I.G.); mpetukh@clemson.edu (M.P.)

**Keywords:** missense mutation, energy calculation, folding free energy, MM/PBSA method

## Abstract

Folding free energy is an important biophysical characteristic of proteins that reflects the overall stability of the 3D structure of macromolecules. Changes in the amino acid sequence, naturally occurring or made *in vitro*, may affect the stability of the corresponding protein and thus could be associated with disease. Several approaches that predict the changes of the folding free energy caused by mutations have been proposed, but there is no method that is clearly superior to the others. The optimal goal is not only to accurately predict the folding free energy changes, but also to characterize the structural changes induced by mutations and the physical nature of the predicted folding free energy changes. Here we report a new method to predict the Single Amino Acid Folding free Energy Changes (SAAFEC) based on a knowledge-modified Molecular Mechanics Poisson-Boltzmann (MM/PBSA) approach. The method is comprised of two main components: a MM/PBSA component and a set of knowledge based terms delivered from a statistical study of the biophysical characteristics of proteins. The predictor utilizes a multiple linear regression model with weighted coefficients of various terms optimized against a set of experimental data. The aforementioned approach yields a correlation coefficient of 0.65 when benchmarked against 983 cases from 42 proteins in the ProTherm database. Availability: the webserver can be accessed via http://compbio.clemson.edu/SAAFEC/.

## 1. Introduction

Folding free energy is an important characteristic of proteins that is directly associated with the stability of the corresponding macromolecule. Changes in the amino acid sequence of a protein, resulting from non-synonymous single nucleotide polymorphism (nsSNP) or artificially designed mutations may alter macromolecular stability [1]. Mutations affecting protein stability are frequently linked to various human diseases [2,3,4,5], including Alzheimer’s disease [6], Salt & Pepper syndrome [7], Snyder-Robinson syndrome [8,9], Rett syndrome [4,8,9,10], and many others [11,12,13]. While folding free energy changes can be determined experimentally, these techniques are usually costly and time consuming. Therefore, developing *in-silico* methods to predict stability changes has been of great interest in the past few decades [5,14].

Various approaches have been proposed to predict folding free energy changes due to missense mutations [5,14,15]. These methods are grouped into two classes: structure based and sequence based. Sequence based methods, like I-Mutant [16], utilize the amino acid sequence of proteins along with neural networks, support vector machines, and decision trees to predict changes in the folding free energy. While such methods can achieve high accuracy in discriminating disease-causing and harmless mutations, they do not predict structural changes caused by the mutation. Alternatively, structure based methods, which include FoldX [17,18], Eris [19], PoPMuSiC [20,21], and others [22], can either only predict whether or not a mutation stabilizes or destabilizes a given structure, or they can output the magnitude of folding free energy change as well. It is additionally useful to reveal the structural changes associated with mutation [23]. These different approaches make predictions that correlate with experimental values to varying degrees, but comparing predictors is complicated because they use different databases of structures for training. In all cases, it is desirable to improve the accuracy of predictions and to provide additional information on the structural changes caused by mutation and the contribution of individual energy terms to the predicted folding free energy change [24,25].

Here we report on a new method to predict the Single Amino Acid Folding free Energy Changes (SAAFEC) based on a knowledge-modified Molecular Mechanics Poisson-Boltzmann (MM/PBSA) approach and a set of terms delivered from the statistical study of physicochemical properties of proteins. The predictor was tested against a dataset containing 983 mutations from the ProTherm database [26,27,28]. We developed a web application utilizing our approach that allows for large-scale calculations.

## 2. Results

Our goal was to develop a fast and accurate structure-based approach for predicting folding free energy changes (ΔΔG) caused by missense mutations. In addition, our predictor was intended to be capable of performing large-scale calculations in a reasonable amount of time. Our method uses a multiple linear regression model to combine a weighted MM/PBSA approach with knowledge-based terms to increase correlation to experimental ΔΔG values from the ProTherm database. We describe the investigation of various parameters and the determination of the weighted coefficients below. We outline (a) the work done to find the optimal parameters for the MM/PBSA method; (b) the statistical analysis performed to find structural features that can be used as flags to predict if a mutation is supposed to cause large or small change of the folding free energy; and (c) the optimization of the weight coefficients. Finally, we provide benchmarking results.

### 2.1. Optimizing MM/PBSA Parameters

#### 2.1.1. Determining Optimal Minimization Steps for the NAMD Protocol and for Finding the Dielectric Constants of the Generalized Born (GB) Model

As described in the Materials and Methods section, we subjected both the wild type (WT) and the mutant (MT) structures to energy minimization to relax structural imperfections and to allow for energy analysis. We performed the minimization of the structures using the Generalized Born implicit model in NAMD [20]. We tried and tested several different dielectric constants for the GB model. We found that a dielectric constant of 80 for the solvent and 1 for the protein gave the highest correlation between predicted folding free energy changes and the experimental data. Note that a similar observation was made in another study [29]. Furthermore, we tested several numbers of equilibrium steps, and we found that 3000 minimization steps when calculating the structures were necessary to achieve the greatest correlation to the experimental data of folding free energy changes. When we tested higher values, we found that more steps require additional computational power and time without significant improvement in the correlation coefficient. Based on these observations, we used 3000 energy minimization steps in the SAAFEC protocol.

#### 2.1.2. Determining the Dielectric Constants for Various Regions in Protein Structure for the Poisson-Boltzmann (PB) Solvation Energy Calculations

**Figure 1:**
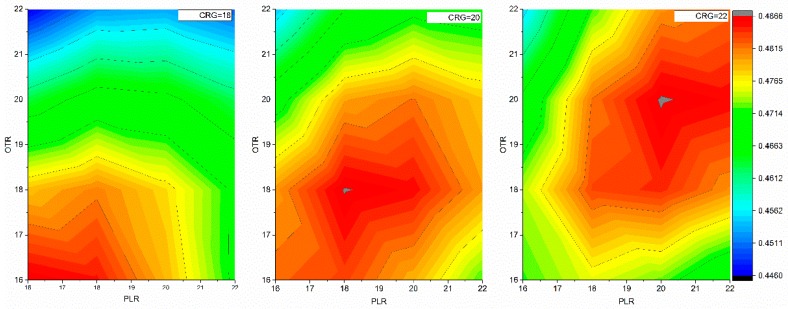
The effect of different dielectric constants for charged (CRG), polar (PLR), and other (OTR) amino acid groups on the correlation coefficient. The value of the correlation coefficient is represented in color, and the color scheme is on the right of the figure. We performed multiple linear regression analysis of predicted folding free energy against experimental data using EE, VE, and SP energy terms.

We initially tried to use the dielectric constants that we had used to minimize the structures when determining the electrostatic components of the energy. However, this approach led to a poor correlation between the predicted folding free energy changes and the experimental data. Our previous work on predicting folding and binding free energy changes indicated that various groups of amino acids sharing common biophysical characteristics can be modeled using different dielectric constant values [29,30]. We demonstrated that the predicted folding free energy changes due to mutations involving charged residues correlate best with experimental data when one uses different dielectric constants for charged, polar and other type of amino acids, respectively [30]. Here, we extended this approach and systematically varied the value of the dielectric constant for charged, polar, and all other groups. Then we performed a linear regression between the predicted folding free energy changes and the experimentally determined ones to obtain the corresponding correlation coefficient. Our goal was to find the optimal dielectric constant values that maximize the correlation coefficient between predicted and experimental folding free energy changes. We made the predictions using only EE, VE, and SP energy terms (see Materials and Methods for details). Figure 1 shows the contour maps of the correlation coefficients for various dielectric constant combinations. The dielectric constant value for the charged groups is fixed in each map, while the dielectric coefficients vary for the polar group on the x-axis and the other group on the y-axis. The gray area on the contour map indicates the highest correlation coefficient. We found that the correlation coefficient was maximized when the dielectric constants were 22 for charged groups, 20 for polar, and 20 for other.

### 2.2. Statistical Analysis

**Figure 2:**
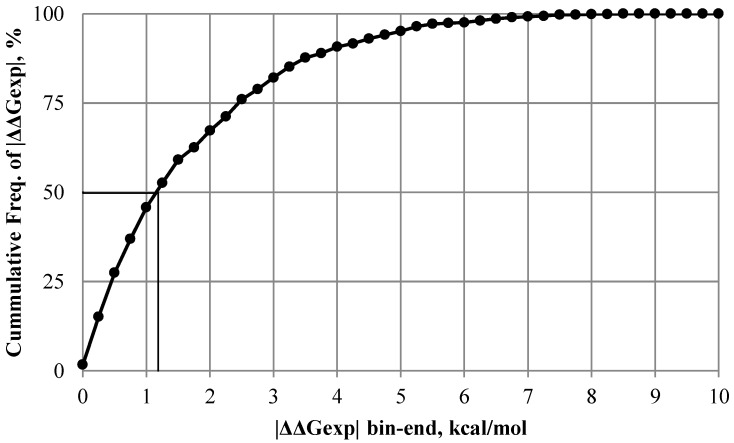
The distribution of the absolute values of the ΔΔGexp within sDB (statistical dataset).

Figure 2 illustrates the distribution of experimentally measured values of the change in folding free energy due to single amino acid substitution in sDB (statistical dataset, see Materials and Methods for details regarding the selection of the dataset). The extreme values vary from −8.33 to +8.64 kcal/mol. We found that in more than half of the total 1262 cases the mutation significantly affects ΔΔG (>1 kcal/mol by absolute value). However, using this frequently used cut-off of 1 kcal/mol [29,31,32] does not split the database into two equally populated datasets, since the number of cases causing a large effect (>1 kcal/mol by absolute value) is bigger than those causing a small effect. To account for such an inequality, a small correction will be suggested in developing flags in predicting if a mutation will cause large or small effect on the folding free energy. It will be demonstrated that these two classes of mutations should better be treated differently (different weight coefficients, see Equations (11) and (12)). For this purpose, we re-evaluated the experimental data and then investigated the probability that large/small ΔΔG are associated with structural features.

One may expect that the magnitude of the effect of mutations on the proteins’ stability can be associated with different biophysical properties, including structural and sequence characteristics. To test such a possibility, we introduced four flags to predict the binary magnitude (small or large) of the effect of mutations on the folding free energy by evaluating the corresponding probabilities of the type of substitution, as well as the location and secondary structure element (SSE) where the mutation takes place. To do that, we split the whole sDB into two subsets: one set with “small effect” (|ΔΔGexp|<1 kcal/mol), and another with “large effect” (|ΔΔGexp|≥1 kcal/mol). Then, the probability (P) of the mutation to cause a large or small effect will be associated with four flags: (flag 1) WT type residue (P(X→any), where the residue of interest, *X*, is substituted with any type of residue); (flag 2) MT type residue (P(any→Y), where any type of residue is substituted with the residue of interest, *Y*); (flag 3) the location of mutation site (P(loc)); and (flag 4) the SSE where the mutation site is located (P(SSE)). These probabilities will be estimated as a ratio of the number of cases causing “large effect” set ($M_{l\arg e}$) divided by the total number of cases (M) in sDB (Equation (1)). As mentioned above, we chose 1 kcal/mol to be the cut-off between the two subsets (large/small effects sets) because it is the default value used in literature [21,31,32]. However, when using this cut-off, the number of cases in the “small” effect set is smaller than in the “large effect” set by 6.9% as mentioned above. Because the number of cases in both sub-sets is different, we calculated the unbiased value probabilities by using this correction term, ΔN (Equation (1)). It allows outputting the probabilities assuming that both datasets are equal in size. Thus, ΔN=0.069 for sDB.

(1) $$P=\frac{M_{\mathrm{large}}}{M}\cdot (1+\Delta N)$$

The corresponding probabilities for mutation types for both WT and MT are shown in Table 1 and for mutation site location and SSE are shown in Table 2. We found that the highest probability of causing “large effect” was for hydrophobic and aromatic residues. For soluble proteins, as the proteins in the ProTherm dataset, this is likely due to the tendency of hydrophobic residues to be in the hydrophobic core of the proteins. Their substitution, particularly with polar or charged residues, could significantly destabilize the protein. Aromatic residues also participate in stabilizing the structure of proteins by maintaining specific interactions. Thus a substitution involving one of the partners that contributes to the “stacking effect” might affect the whole structure. Conversely, the higher probability of charged residue substitutions to cause “small effect” may be due to their tendency to be on the surface of the protein and be exposed to the solvent.

We found that mutations in helix-strand regions have the tendency to cause a large change in folding free energy (Table 2). Coil and turn SSE are often found in the solvent exposed area of the proteins, and thus their substitution is unlikely to affect the proteins’ stability. Similarly, mutation of the solvent exposed residues would be tolerable for the proteins’ stability with the probability of causing “large effect” being only 31%. Mutations in the buried region of the proteins, on the other hand, are expected to induce large conformational rearrangements.

We proceeded further with the probabilities listed in Table 1 and Table 2 at the next step, considering the possibility that they may be correlated. To address such an effect, we altered the $\left|\Delta \Delta G_{k}\right|$ with respect to each of the four flags for each case in sDB by applying the following formula:

(2) $$\left|\Delta \Delta G_{k}^{altered}\right|(\mathrm{for}P_{i}\mathrm{set})=\left\{\begin{array}{c} \frac{2}{3}\cdot \sum_{j=1,i\neq j}^{4}P_{j}\cdot \left|\Delta \Delta G_{k}\right|,\left|\Delta \Delta G_{k}\right|<1\\ \frac{2}{3}\cdot \sum_{j=1,i\neq j}^{4}(1-P_{j})\cdot \left|\Delta \Delta G_{k}\right|,\left|\Delta \Delta G_{k}\right|\geq 1 \end{array}\right\}$$

where *P_j_* stands for: $P_{1}=P(X\to any)$, $P_{2}=P(any\to Y)$, $P_{3}=P(loc)$, and $P_{4}=P(SSE)$. These alterations were done for each entry in sDB and for each of the flags. Thus, for example, if one applies Equation (2) for altering the $\left|\Delta \Delta G_{k}\right|$ probability of flag “*k*” using the other three flags’ probabilities, and if the other three probabilities are 0.5, *i.e.*, the initial statistical analysis of sDB shows that the type of mutation has an equal chance to cause large and small effect, this will result in no alteration: $\left|\Delta \Delta G_{k}^{altered}|=|\Delta \Delta G_{k}\right|$.

The resulting set of $\left|\Delta \Delta G^{altered}\right|$ is termed altered dataset, and subsequently was used to recalculate the probabilities $P^{\prime }$ (Table 1 and Table 2). These altered probabilities and classifications will be used to improve the performance of the SAAFEC method. Thus the resulting probability of the mutation to cause a “large effect” in the proteins’ stability is calculated as the average of four flags:

(3) $$P=\frac{P\left(X\to any\right)+P\left(any\to Y\right)+P\left(loc\right)+P\left(SSE\right)}{4}$$

Based on the resulting probability (received from the knowledge of only the type of WT and MT residues, the location of the mutated site, and the SSE of the mutated site), we divided the tDB (the training dataset, see Materials and Methods for details) into two: (a) cases that are predicted to have large effect on the protein stability, if P≥0.5; and (b) cases that are predicted to have small effect on the protein stability, if P<0.5. In this step, we take advantage of estimated probabilities to enhance the prediction made by the SAAFEC algorithm.

### 2.3. Optimizing Weight Coeficients and Benchmarking Results

Based on previous work and knowledge about the biophysical characteristics of proteins, we tested several knowledge-based energy terms and used them to improve the correlation coefficient between values we predicted using Equation (6) with the corresponding MM/PBSA and KB components and experimental ΔΔG values. We obtained two formulas, Equations (11) and (12), based on the analysis of the correlation coefficient (see Materials and Methods). The decision to apply Equation (11) or Equation (12) is based on the probabilities described by Equation (3).

**Figure 3:**
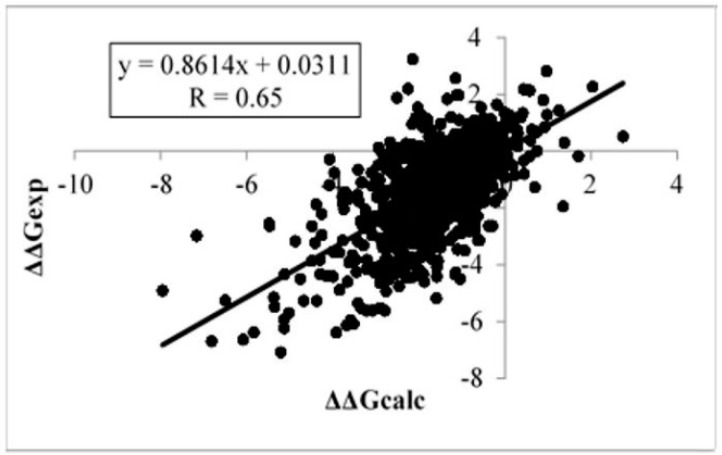
Correlation between experimental data and values calculated with the Single Amino Acid Folding free Energy Changes (SAAFEC) approach of the change in folding free energy due to single point amino acid mutations.

Table 3 shows the results of the multiple linear regression approach used to correlate the calculated and experimental folding free energy changes and the corresponding energy terms. We used a backwards elimination technique to leave only the energy components that contributed significantly (small *p*-value and large weight coefficient). The resulting weights and *p*-values from the multiple linear regression are presented in the table. The optimization resulted in a final correlation coefficient of 0.65 (*R*_final_). This is significantly better than the correlation coefficients for “small” (*R* = 0.36) and “large” (*R* = 0.62) effect cases (Table 3). When we did not use the flags described above, the correlation coefficient was *R* = 0.62 (Figure 3).

Some of the knowledge-based energy terms may overlap with the MM/PBSA energy components, or they might be closely related themselves. However, the p-values reported in Table 3 indicate that each of the selected energy terms significantly contributes to the reported correlation coefficient.

Continuing with benchmarking, we evaluated the method’s performance for specific groups of mutations based on: (a) SSE—“helical/strand” (HS, HH, SS), and “coil” (CC, CT, TT) regions; (b) location; and (c) amino acid types—Ala-scanning database (Any→A), and the cases when the bulky residues (R, F, W, and Y) are substituted with a smaller size residue (A, S, G, and V). Arginine (R) is included in the bulky residues list because of its long side chain. The parameters of the linear regression analysis between experimental (*y*-axis) and calculated (*x*-axis) values of (number of cases, correlation coefficient, slope and Y-intercept) are shown in Table 4.

We found that the effect of mutations occurring in “helical/strand” regions on the change of folding free energy can be predicted with higher accuracy (*R* = 0.67), than the one in the “coil” region (*R* = 0.57) (Table 4). The precision of the SAAFEC method also depends on the location of the mutated residue. Thus, the highest correlation coefficient between experimental and calculated values is reached when both WT and MT residues are partially exposed to the solvent (*R* = 0.64). However, we found that the correlation is lower for totally exposed residues (*R* = 0.37). However taking into consideration the fact that in the majority of cases, when both WT and MT residues are exposed, the mutation does not significantly influence the proteins’ stability (the 25th, 50th, and 75th percentiles of the absolute energy change are 0.26, 0.60, and 1.02 kT respectively), one may explain the small value of correlation coefficient by the fact that experimental values are scattered around zero.

The “Ala-scanning” technique is widely used to determine “hot-spots” of proteins. Thus, the sequential mutation of a residue to a small, hydrophobic Ala and estimation of the change of the protein stability determines the impact of each WT residue on the protein fold stability. The SAAFEC method shows good agreement with experimental values (*R* ≈ 0.7), calculated for 301 Ala cases in tDB (Table 4).

The SAAFEC algorithm also shows a high correlation coefficient (*R =* 0.67) for the cases involving a mutation of large residues (Arg, Phe, Tyr, and Trp) to a small one (Ala, Gly, Ser, and Val) (Table 4).

Predicting the effect of mutation on protein folding free energy can be used to discriminate between disease-causing and harmless mutations, and thus has implications to human health [2,4,5,14]. Studies have shown high correlation between the degree of harmfulness of mutations and the effect of mutations on the protein stability [9,21,23] (see SI for details, Table S1). We have found that the SAAFEC method achieves high accuracy and high sensitivity. Matthew correlation coefficient of 0.833 (see SI, Table S2 for more details) indicates that our computational method can potentially be used to estimate the harmfulness of mutations.

## 3. Discussion

This work reports a new method (SAAFEC) and a webserver to predict the folding free energy changes caused by amino acid mutations. We benchmarked the approach against 983 experimental data-points and achieved a correlation coefficient of 0.652, which is similar to the performance of other leading predictors (see SI, Table S5). However, SAAFEC not only predicts the folding free energy changes, but also reports the changes of the corresponding energy components and provides energy-minimized structures of both the WT and the MT. This allows the users to carry out further structural analysis of the effects of mutations.

## 4. Materials and Methods

Here, we describe the method of calculating the change of the folding free energy caused by amino acid substitution. It is based on two distinctive components: (a) Molecular Mechanics Poisson-Boltzmann Surface Accessibility (MM/PBSA) energies and (b) Knowledge-Based (KB) terms. The combined usage of MM/PBSA and KB terms makes the method distinctively different from the existing ones. The MM/PBSA and KB terms are combined in a linear equation with corresponding weight coefficients. The weight coefficients are then optimized against experimental data taken from the ProTherm database [26]. Below we outline the selection of experimental data, the structural features taken into account, the simulation protocol for MM/PBSA, and various KB terms used in the equations.

### 4.1. Construction of the Experimental Dataset

A dataset containing experimentally measured values of folding free energy changes due to single point amino acid mutations was constructed from the ProTherm database [26]. The initial dataset was subjected to a validity check, because some of the entries are reported several times and the reported folding free energy changes are not the same. Thus, at the beginning the set was screened for repeating values and only one representative was retained. The data was further purged to eliminate cases where the experimental pH value was below 5 or above 9. When several experimental values were reported for the same mutation in the same protein, and the experimental data variation was less than 0.1 kcal/mol, the entries were fused, and the average was used. Entries that did not satisfy this condition were deleted. This dataset (49 proteins, 1262 mutations) was used for statistical analysis (sDB). We further pruned the data set to leave only cases, where the X-ray crystallographic structures of the protein did not contain ligands. This dataset (42 proteins, 983 mutations) was used for testing the proposed algorithm (tDB).

### 4.2. Degree of Burial

To determine the degree of burial of a residue in the protein, we calculated its relative solvent accessible surface area (rSASA) with NACCESS software [24]. Here, we distinguished three possible degrees of burial: buried (B, rSASA = 0), partially exposed (PE, Rsasa ≤ 0.25 and rSASA > 0), and exposed (E, rSASA > 0.25) Thus, the residues characterized as PE and E are accessible from the water, while the residues defined as B are completely buried inside the protein (see SI, Table S3).

### 4.3. Secondary Structure Element

We distinguished five groups of the secondary structure elements (SSE) in which a residue can be located: helix (H), coil (C), turn (T), strand (S), and bridge (B). The SSE was determined for the residue in both the wild type and the mutant type proteins with STRIDE software [33].

### 4.4. Simulation Protocol

The initial structures of the proteins were obtained from the Protein Data Bank (in PDB file format) [26]. The structures were manually screened and only biological units were retained for further analysis. We used the profix module of the Jackal package with default parameters and the “heavy atoms model” option to reconstruct missing proteins atoms and residues. We obtained the mutant type protein structure (MT) by substituting the wild-type residue with the mutant one using the scap module of the Jackal package [34,35]. To maintain consistency, we mutated the wild-type residue with the same one following the above-mentioned procedure to obtain the wild-type protein structure (WT). The parameters used with scap included the CHARMM 22 force field, thorough side chain refinement, the default number of initial structures tried, and the default side-chain rotamer library provided by the software. We generated missing hydrogen atoms for both WT and MT structures by applying the VMD (version 1.9.1) software [36,37] with the CHARMM27 force field parameters. The structures of both WT and MT proteins were then subjected to independent refinement with NAMD (version 2.9) software [38,39]. Both of the structures (WT, and MT) were then minimized using the Generalized Born implicit solvent model in NAMD. The structures were relaxed for 3000 steps utilizing the CHARMM27 force field parameters, as well as a dielectric constant of 80 for the solvent and 1 for the protein. Three-residue segments (WT_residue_ and MT_residue_ that are consecutive residues with the mutated one in the center) were isolated from the energy-minimized WT and MT structures respectfully to represent the unfolded state of the proteins. This approach, which was introduced before [22,40], was tested against different segment length from 3 up to 11, and it was shown that 3 residues length is optimal [22]. These four structures (WT, MT, WT_residue_, MT_residue_) were then used to calculate all energy components.

### 4.5. Free Folding Energy Calculations

The folding free energy for WT and MT can be calculated as following:

(4) $$\Delta G=G_{\left(folded\right)}-G_{\left(unfolded\right)}$$

Thus, the change in folding free energy of the protein due to mutation is:

(5) $$\Delta \Delta G_{\left(mutation\right)}=[G_{\left(folded\right)}MT-G_{\left(unfolded\right)}MT]-[G_{\left(folded\right)}WT-G_{\left(unfolded\right)}WT]$$

Following our previous work [16], the unfolded protein (both WT and MT) can be considered to be comprised of two parts: (a) a three residue segment containing the mutation and one residue on either side; and (b) all other residues. Therefore, we can assume that the mutation only affects residues in the immediate vicinity of the mutation site and does not affect the rest of the protein. The energy of the non-affected fraction of the unfolded protein is then identical for WT and MT and cancels out. Therefore the change in folding free energy can be calculated as:

(6) $$\Delta \Delta G_{\left(mutation\right)}=[G_{\left(folded\right)}MT-G_{3}MT]-[G_{\left(folded\right)}WT-G_{3}WT]$$

where *G*_3_ is the free energy of the 3-residue segment.

The SAAFEC method calculates the change in folding free energy (ΔΔG) of proteins caused by single amino acid substitution. It utilizes the MM/PBSA approach in a combination with other knowledge-based parameters. The value of ΔΔG was calculated through the linear combination of multiple energy terms. The weight of each energy terms was estimated by applying a multiple linear regression analysis with backwards elimination against experimentally determined values of ΔΔG (taken from tDB). Only significant terms, with *p*-value smaller than 0.05, were taken into account. The individual terms used in the SAAFEC approach are described in detail below.

### 4.6. The MM/PBSA-Based Components of the SAAFEC Method

The MM/PBSA-based component of the SAAFEC method is a linear function combining four energy terms:

(7) $$\Delta \Delta G^{MM/PBSA}=w_{0}+w_{1}*\Delta IE+w_{2}*\Delta \Delta VE+w_{3}*\Delta \Delta SP+w_{4}*\Delta \Delta \mathrm{EE}$$

where *w*_k_ are weight coefficients that will be optimized against experimental data. Below we outline the energy components in Equation (7).

ΔIE (change in internal energy) was calculated as the difference between the internal energies (IE = sum of BOND, ANGLE, DIHED, and IMPRP) of the WT and MT structures. The IE was calculated via NAMD by applying a 1-step energy minimization procedure with an implicit solvent model. The same was done for the 3-residue segments.

ΔΔVE (the difference of the change in Van der Waals energy) was calculated as the difference of VE terms for WT, MT, WT_residue_, and MT_residue_ structures. VE was calculated via NAMD by applying a 1-step energy minimization procedure with an implicit solvent model.

ΔΔEE (the difference of the change in Coulombic energy) and ΔΔSP (the difference of the change in the polar component of solvation energy) energy components were calculated with DelPhi software package [29] using the following parameters: linear Poisson-Boltzmann solver, scale 1 grids/Å, perfil 70% and an external dielectric constant of 80. The initial selection of an internal dielectric constant value of 1, as used during the NAMD minimization protocol, proved undesirable as the correlation coefficient of the multiple linear regression was poor (see Results). Based on previous work done, it was determined that different dielectric constants can be assigned to different amino acid groups to mimic small structural changes [29,30]. In the SAAFEC approach, three different groups of amino acids were used, each with a different dielectric constant (ε1, ε2, and ε3). Charged residues (R,K,H,D,E) were modeled using ε1, polar residues (S,T,N,Q,Y) using ε2, and all others using ε3. Different combinations of dielectric constants values were used in addition to the corresponding regression coefficients to create the contour maps mentioned in the Results section. The polar component of the solvation energy was calculated as the difference of ΔΔSP terms for WT, MT, WT_residue_, and MT_residue_ structures, using the “corrected reaction field energy” module of DelPhi. ΔΔEE was again calculated using DelPhi in a similar approach to ΔΔSP.

### 4.7. The Knowledge-Based Components of the SAAFEC Method

The algorithm applies different knowledge-based formulas depending on the expected folding free energy change caused by a mutation (see Results section). Two classes are considered: a mutation causing small and a mutation causing large change of the folding free energy. Various combinations of KB terms were tested and the corresponding correlation coefficient between predicted and experimental folding free energies was calculated. This was done separately for the cases causing small and large effect. As a result of such an analysis, two KB formulas (Equations (8) and (9)) were obtained.

Thus, the knowledge-based components for cases expected to cause a small effect were calculated according to the following equation:

(8) $$\Delta \Delta G^{KB}=w_{5}*\Delta S_{sum}+w_{6}*\Delta \Delta IE$$

The knowledge-based components for mutations expected to cause large effect were calculated according to the following equation:

(9) $$\Delta \Delta G^{KB}=w_{5}*\Delta S+w_{6}*\Delta S_{sum}+w_{7}*SAS_{MT}+w_{8}*\frac{SN}{SAS_{MT}}$$

Equations (8) and (9) account for four additional terms: entropy of a single side chain (*S*), the sum of the side chain entropies over all residues (*S_sum_*), solvent accessible surface area of the MT (*SAS_MT_*), and the non-polar component of solvation energy normalized to the solvent accessible surface area of the mutant ($\frac{SN}{SAS_{MT}}$). Furthermore, the ΔΔIE term (double change in internal energy) was calculated as the difference between the internal energies (IE = sum of BOND, ANGLE, DIHED, and IMPRP) of the WT and MT and the corresponding WT_residue_, and MT_residue_ structures, *i.e.*, ΔΔIE = ΔIE(MT-WT) − ΔIE(MT_residue_-WT_residue_). The IE was calculated via NAMD by applying a 1-step energy minimization procedure with an implicit solvent model.

The change in entropy of the individual residues (*S*) was calculated using an empirical formula developed in previous work [29]. The entropies are based on the maximum number of side chain rotamers (R) [41] (see SI for more information, Table S4). The formula assumes that an exposed residue (relative solvent accessible surface area equal to one) can access all rotamers, while a buried one (relative solvent accessible surface area equal to zero) can adopt only one rotamer.

(10) S=ln[rSASA*(R−1)+1]

### 4.8. Combining MM/PBSA-Based and Knowledge-Based Terms

The statistical analysis showed that mutations resulting in “large” and “small” changes of the folding free energy should be modeled with different energy terms and different weight coefficients. Thus, combining Equation (7) with Equations (8) and (9), the corresponding formulas are provided below, respectively.

The final formula for mutations expected to cause small effect is as follows:

(11) $$\Delta \Delta G=w_{0}+w_{1}*\Delta IE+w_{2}*\Delta \Delta VE+w_{3}*\Delta \Delta SP+w_{4}*\Delta \Delta EE+w_{5}*\Delta S_{sum}+w_{6}*\Delta \Delta IE$$

The final formula for cases expected to cause large effect is as follows:

(12) $$\Delta \Delta G=w_{0}+w_{1}*\Delta IE+w_{2}*\Delta \Delta VE+w_{3}*\Delta \Delta SP+w_{4}*\Delta \Delta EE+w_{5}*\Delta S+w_{6}*\Delta S_{sum}+w_{7}*SAS_{MT}+w_{8}*\frac{SN}{SAS_{MT}}$$

## 5. Web Server Architecture

### 5.1. General Description

The SAAFEC web server is comprised of a client interface implemented using regular HTML and a server back end that submits the user input for processing on the Palmetto cluster. The back end is implemented using PHP server scripting language while a python script executes on the web cluster and performs the various energy calculations.

### 5.2. User Interface

This is the initial page seen by the client. The user specifies several parameters and uploads a PDB file of the desired structure. The user then specifies the location of the residue. The next job parameter is the type of residue that is present in the structure before the mutation, and the type of residue that must be substituted. Finally, the user submits the name of the chain containing the desired residue location and an email address. The email address is necessary because the user will receive an email with a link that can be used to download the results from the job.

### 5.3. Back End

The back end of the server handles the requests of the user (parameters entered, and uploaded PDB) and submits the job on the Palmetto cluster. PHP commands are used to transfer the parameters and structure to the cluster and submit the job. The job executes a calculation method implemented using Python that generates a results file containing individual energy terms and a free folding energy value. PHP server commands are then used again to transfer the results to another server from which the user can access the results.

### 5.4. Results Page

Upon completion of the job, assuming valid input from the user, the results are stored on the web server and an email with a link is sent to the client. The results include the free folding energy value calculated using the SAAFEC method as well as all of the individual energy terms used to calculate the value.

## Figures and Tables

**Table 1:** The probabilities of the wild type (WT) and mutant type (MT) residues to cause “large effect”. The probability (P) values were calculated using unperturbed (original) |ΔΔG|. The *P*′ values were calculated using altered |ΔΔG^altered^| based on Equation (2).

	WT			MT		
AA	Total Cases	P(X→any)	$P^{\prime }(X\to any)$	Total Cases	P(any→Y)	$P^{\prime }(any\to Y)$
A	91	0.60	0.56	374	0.54	0.53
C	7	0.69	0.55	36	0.44	0.51
D	52	0.48	0.50	34	0.58	0.63
E	93	0.44	0.48	43	0.62	0.64
F	52	0.72	0.69	85	0.45	0.41
G	79	0.52	0.57	167	0.68	0.69
H	41	0.36	0.37	10	0.78	0.79
I	103	0.66	0.63	65	0.40	0.32
K	114	0.22	0.20	32	0.47	0.45
L	92	0.83	0.80	69	0.33	0.25
M	23	0.58	0.59	15	0.43	0.45
N	40	0.62	0.60	19	0.65	0.67
P	42	0.35	0.36	16	0.40	0.36
Q	25	0.25	0.30	41	0.26	0.30
R	37	0.51	0.49	20	0.62	0.53
S	40	0.30	0.21	55	0.60	0.53
T	86	0.50	0.46	39	0.74	0.70
V	141	0.61	0.59	107	0.58	0.52
W	23	0.81	0.86	17	0.44	0.45
Y	81	0.67	0.66	18	0.58	0.65

**Table 2:** The probabilities of the type of the location and the secondary structure element (SSE) of the mutated site to cause “large effect”. The *P′* values are calculated based on the distribution of altered |ΔΔG| (see Equation (2)). If the total number of cases is less than 5, we assign the probability to be 0.5. See the Materials and Methods section for the definitions of SSE Type: BB, CC, CH, CS, CT, HH, HS, HT, SS, ST, and TT.

Location				SSE			
Location Type	Total Cases	*P*(loc)	*P′*(loc)	SSE Type	Total Cases	*P*(SSE)	*P′*(SSE)
B-B	102	0.74	0.70	BB	14	0.26	0.27
B-PE	132	0.78	0.76	CC	182	0.47	0.45
E-E	457	0.31	0.29	CH	6	0.81	0.65
E-PE	130	0.56	0.55	CS	8	0.59	0.61
PE-PE	441	0.65	0.65	CT	6	0.15	0.16
‒	‒	‒	‒	HH	378	0.55	0.53
‒	‒	‒	‒	HS	1	0.50	0.50
‒	‒	‒	‒	HT	2	0.50	0.50
‒	‒	‒	‒	SS	455	0.63	0.61
‒	‒	‒	‒	ST	2	0.50	0.50
‒	‒	‒	‒	TT	208	0.39	0.38

**Table 3:** The optimized weights and the corresponding *p*-values of the multiple linear regression analysis between calculated and experimental values of the change of folding free energy. The correlation coefficient R is reported separately for “small” and “large” effect cases. The bottom line, the R_final_, is reported for two cases: on the right for the entire database without distinguishing the cases of “small” and “large” effect, and on the left applying Equation (3) to predict the corresponding probabilities and then to apply Equations (11) and (12), respectively. The correlation coefficient in parentheses is obtained via 5-fold cross-validation.

	Weight, Small	*p*, Small	Weight, Large	*p*, Large	Weight, All	*p*, All
Y-intercept	−7.44 × 10^−1^	0.00 × 10^0^	−2.27 × 10^0^	0.00 × 10^0^	−1.58 × 10^0^	0.00 × 10^0^
IE	9.28 × 10^−2^	1.36 × 10^−2^	‒	‒	‒	‒
EE	5.93 × 10^−1^	3.37 × 10^−7^	8.54 × 10^−1^	0.00 × 10^0^	8.93 × 10^−1^	0.00 × 10^0^
VE	7.51 × 10^−2^	2.03 × 10^−4^	1.63 × 10^−1^	0.00 × 10^0^	1.69 × 10^−1^	0.00 × 10^0^
SP	4.53 × 10^−1^	5.14 × 10^−8^	6.32 × 10^−1^	0.00 × 10^0^	6.68 × 10^−1^	0.00 × 10^0^
S	‒	‒	4.07 × 10^−1^	4.18 × 10^−2^	4.85 × 10^−1^	1.03 × 10^−3^
HYDR	‒	‒	‒	‒	−1.57 × 10^0^	9.63 × 10^−3^
Ssum	−1.26 × 10^−1^	1.99 × 10^−5^	−6.55 × 10^−1^	4.05 × 10^−4^	−6.67 × 10^−1^	2.24 × 10^−6^
SAS_MT_	NA	NA	9.36 × 10^−5^	1.10 × 10^−4^	−5.46 × 10^1^	2.88 × 10^−3^
SN/SAS_MT_	NA	NA	−7.71 × 10^−1^	6.84 × 10^−3^	−2.78 × 10^1^	4.77 × 10^−2^
R	0.36	‒	0.62	‒	‒	‒
#Poins	426	‒	558	‒	984	‒
R final	0.65 (0.61)	‒	‒	‒	0.62	‒

**Table 4:** Performance of Single Amino Acid Folding free Energy Changes (SAAFEC) method in predicting the effect of specific groups of mutations.

		Cases	R	Slope	Y-Intercept	Min	Max
SSE	HS, HH, SS	652	0.67	0.92	0.07	0.00	7.09
	CC, CT, TT	310	0.58	0.67	−0.08	0.00	6.13
Location	B-B	83	0.60	0.86	−0.02	0.02	7.09
	B-PE	99	0.62	0.93	0.08	0.00	6.71
	PE-PE	308	0.64	0.84	0.04	0.00	6.39
	E-PE	102	0.52	0.80	0.01	0.03	6.39
	E-E	396	0.37	0.64	−0.12	0.00	4.51
Residues	Any→A	301	0.69	0.89	0.18	0.00	5.54
	Large (RFWY)→Small (AGSV)	67	0.67	0.86	0.06	0.04	6.39

## Supplementary Materials

Supplementary materials can be found at http://www.mdpi.com/1422-0067/17/4/512/s1. The sDB and the tDB databases are available online at http://compbio.clemson.edu/SAAFEC/databases/sDB,%20tDB.xlsx.
