# Supplementary material for: SAAFEC: Predicting the Effect of Single Point Mutations on Protein Folding Free Energy Using a Knowledge-Modified MM/PBSA Approach
Source: Int J Mol Sci. 2016 Apr 7;17(4):512. doi: 10.3390/ijms17040512 (PMC4848968; doi:10.3390/ijms17040512)
Supplement: Supplementary file 1 [file ijms-17-00512-s001.pdf]

Supplementary Material: SAAFEC: Predicting the Effect of Single Point Mutations on Protein Folding Free Energy Using a Knowledge-Modified MM/PBSA Approach

Ivan Getov, Marharyta Petukh and Emil Alexov

Typically the accuracy of predictions of disease-causing mutations is evaluated via the ROC parameters adopting particular cut-offs for true and false positives. Several quantities are evaluated with Equations (S1)–(S6) using the relationship between four quantities: true positive (tp), true negative (tn), false positive (fp) and false negative (fn) (definitions are provided in Table S1. The cut-offs of 0.5 and 1.5 kcal/mol are taken from our previous work [29]). The corresponding ROC results are shown in Table S2.

$$accuracy = \frac{tp + tn}{tp + tn + fp + fn} \quad (S1)$$

$$sensitivity = \frac{tp}{tp + fn} \quad (S2)$$

$$specificity = \frac{tn}{tn + fp} \quad (S3)$$

$$precision = \frac{tp}{tp + fp} \quad (S4)$$

$$NPV = \frac{tn}{tn + fn} \quad (S5)$$

$$MCC = \frac{tp \cdot tn + fp \cdot fn}{\sqrt{(tp + fp) \cdot (tp + fn) \cdot (tn + fp) \cdot (tn + fn)}} \quad (S6)$$

Table S1. Four possible relationships between experimental and calculated values of the change of folding free energy.

	True	False
positive	$\left\{\begin{array}{l} \lvert\Delta\Delta Gexp\rvert \geq 1.5 \\ \lvert\Delta\Delta Gcalc\rvert \geq 0.5 \\ sign(\Delta\Delta Gexp) = sign(\Delta\Delta Gcalc) \end{array}\right\}$	$\left\{\begin{array}{l} \lvert\Delta\Delta Gexp\rvert \leq 0.5 \\ \lvert\Delta\Delta Gcalc\rvert \geq 1.5 \end{array}\right\}$
negative	$\left\{\begin{array}{l} \lvert\Delta\Delta Gexp\rvert \leq 0.5 \\ \lvert\Delta\Delta Gcalc\rvert \leq 1.5 \end{array}\right\}$	$\left\{\begin{array}{l} \lvert\Delta\Delta Gexp\rvert \geq 1.5 \\ \lvert\Delta\Delta Gcalc\rvert \leq 0.5 \end{array}\right\}$

Table S2. Calculated ROC quantities. NPV is negative predictive value and MCC is Matthew correlation coefficient.

Accuracy	0.914
Sensitivity	0.965
Specificity	0.837
Precision	0.899
NPV	0.942
MCC	0.833

Further we provide definitions for classifying the mutation sites as buried, partially buried and exposed (Table S3).

Table S3. Description of residue burial in proteins in tDB.

**Buried (B)**	**rSASA = 0**
Partially Exposed (PE)	rSASA ≤ 0.25 and rSASA > 0
Exposed (E)	rSASA > 0.25

The maximum number of rotamers used for the entropy estimation. (Table S4).

Table S4. The maximum number of rotamers per residue.

	**A**	**C**	**D**	**E**	**F**	**G**	**H**	**I**	**K**	**L**	**M**	**N**	**P**	**Q**	**R**	**S**	**T**	**V**	**W**	**Y**
R	1	3	18	54	18	1	36	9	81	9	27	36	2	108	81	3	3	3	36	18

Comparisons of SAAFEC performance with other leading folding free energy changes predictors (Table S5). The results are obtained by taking randomly 100 cases from tDB.

Table S5. Predictors and their performance.

**Predictor**	**Correlation Coefficient (R)**
SAAFEC	0.59
PoPMuSiC	0.34
FoldX	0.42
I-Mutant	0.67
DUET	0.58
